# Angiogenesis PET Tracer Uptake (^68^Ga-NODAGA-E[(cRGDyK)]_2_) in Induced Myocardial Infarction and Stromal Cell Treatment in Minipigs

**DOI:** 10.3390/diagnostics8020033

**Published:** 2018-05-16

**Authors:** Thomas Rasmussen, Bjarke Follin, Jens Kastrup, Malene Brandt-Larsen, Jacob Madsen, Thomas Emil Christensen, Morten Juhl, Smadar Cohen, Karsten Pharao Hammelev, Christian Holdflod Møller, Jens Peter Goetze, Philip Hasbak, Andreas Kjær

**Affiliations:** 1 Department of Clinical Physiology, Nuclear Medicine & PET and Cluster for Molecular Imaging, Department of Biomedical Sciences, Rigshospitalet and University of Copenhagen, Blegdamsvej 9, 2100 Copenhagen, Denmark; drrasmussen@hotmail.com (T.R.); bjarke.follin@regionh.dk (B.F.); malene.brandt-larsen@regionh.dk (M.B.-L.); jacob.madsen@regionh.dk (J.M.); thomas.emil.christensen@regionh.dk (T.E.C.); philip.hasbak@regionh.dk (P.H.); 2 Cardiology Stem Cell Centre, Department of Cardiology, The Heart Centre, Rigshospitalet, University of Copenhagen, 1165 Copenhagen, Denmark; jens.kastrup@regionh.dk (J.K.); morten.juhl@regionh.dk (M.J.); 3 Avram and Stella Goldstein-Goren Department of Biotechnology Engineering and Regenerative Medicine and Stem Cell (RMSC) Research Center, Ben-Gurion University of the Negev, Beer-Sheva 84105, Israel; scohen@bgu.ac.il; 4 Department of Experimental Medicine, University of Copenhagen, 1165 Copenhagen, Denmark; kah@sund.ku.dk; 5 Department of Cardiothoracic Surgery, The Heart Centre, Rigshospitalet, University of Copenhagen, 1165 Copenhagen, Denmark; Christian.moeller.02@regionh.dk; 6 Clinical Biochemistry, Copenhagen University Hospital, Rigshospitalet, 2100 Copenhagen, Denmark; jens.peter.goetze@regionh.dk

**Keywords:** myocardial perfusion, RGD, rubidium, cardiac positron-emission-tomography, angiogenesis, mesenchymal stromal cells, hydrogel, stem cell, myocardial infarction

## Abstract

Angiogenesis is considered integral to the reparative process after ischemic injury. The α_v_β_3_ integrin is a critical modulator of angiogenesis and highly expressed in activated endothelial cells. ^68^Ga-NODAGA-E[(cRGDyK)]_2_ (RGD) is a positron-emission-tomography (PET) ligand targeted towards α_v_β_3_ integrin. The aim was to present data for the uptake of RGD and correlate it with histology and to further illustrate the differences in angiogenesis due to porcine adipose-derived mesenchymal stromal cell (pASC) or saline treatment in minipigs after induction of myocardial infarction (MI). Three minipigs were treated with direct intra-myocardial injection of pASCs and two minipigs with saline. MI was confirmed by ^82^Rubidium (^82^Rb) dipyridamole stress PET. Mean Standardized Uptake Values (SUV_mean_) of RGD were higher in the infarct compared to non-infarct area one week and one month after MI in both pASC-treated (SUV_mean_: 1.23 vs. 0.88 and 1.02 vs. 0.86, *p* < 0.05 for both) and non-pASC-treated minipigs (SUV_mean_: 1.44 vs. 1.07 and 1.26 vs. 1.04, *p* < 0.05 for both). However, there was no difference in RGD uptake, ejection fractions, coronary flow reserves or capillary density in histology between the two groups. In summary, indications of angiogenesis were present in the infarcted myocardium. However, no differences between pASC-treated and non-pASC-treated minipigs could be demonstrated.

## 1. Introduction

The formation of new capillaries or angiogenesis, is considered a vital part of the natural recovery after an ischemic injury and is, therefore, a key factor for post-ischemic repair of the infarcted myocardium [1]. Since angiogenesis is associated with post-infarct remodeling of the left ventricle, and thus prognosis following myocardial infarction, it is of particular interest to be able to noninvasively monitor angiogenesis [2,3]. This might not only permit for a risk stratification of patients following myocardial infarction, but also facilitate development and improvement of new therapies directed towards stimulation of the natural angiogenic response.

Myocardial angiogenesis following myocardial infarction might be focal or non-transmural making it difficult to detect noninvasively, and existing noninvasive imaging methods directed towards the evaluation of angiogenesis have been somewhat limited. 

During angiogenesis, endothelial cells must adhere to one another and to the extracellular matrix in order to form new microvessels and to extend existing ones [4,5]. The cross-talk with the extracellular matrix is mediated by integrins, which are a family of heterodimeric cell surface receptors involved in many cellular processes, including adhesion, migration, proliferation, and survival [6]. Specifically, the α_v_β_3_ integrin has been identified as a critical modulator of angiogenesis. It is highly expressed in activated endothelial cells, and therefore serves as a potential target for directly imaging angiogenesis [5,7,8,9].

The aim of this study was to present data for the uptake of a newly developed angiogenesis positron-emission-tomography (PET) tracer targeted towards α_v_β_3_ integrin (^68^Ga-NODAGA-E[c(RGDyK)]_2_ (RGD)) [7] and correlate it with histology and further to illustrate the differences in angiogenesis in Göttingen minipigs treated with saline or porcine adipose-derived mesenchymal stromal cell (pASC) suspended in alginate hydrogel after induction of acute myocardial infarction.

## 2. Materials and Methods

### 2.1. Study Design

**Figure 1:**
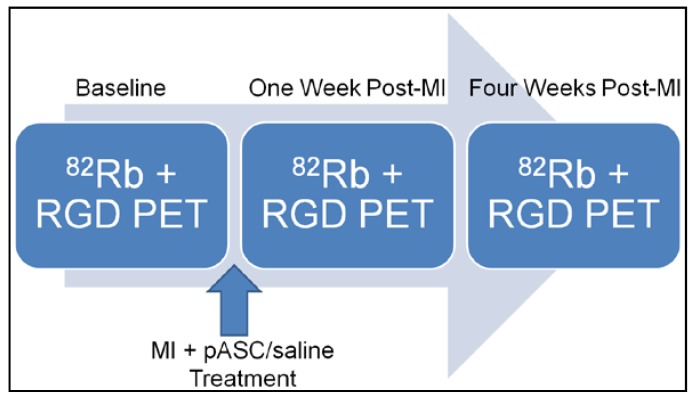
Flowchart showing the time points for ^82^Rb and RGD PET scans, MI induction and pASC/saline treatment. Abbreviations: ^82^Rb: ^82^Rubidium; RGD: ^68^Ga-NODAGA-E[c(RGDyK)]_2_; PET: Positron Emission Tomography; MI: Myocardial infarction; pASC: porcine adipose-derived mesenchymal stromal cell.

Five Göttingen minipigs were used in this study. The study was approved by The National Committee for the Protection of Animals used for Scientific Purposes in Denmark (1 July 2014, Authorization number 2014-15-0201-00191). The infarction model was an acute model of myocardial infarction with permanent ligation and pASC or saline treatment immediately after the infarct induction. The minipigs underwent PET-imaging examining myocardial perfusion (^82^Rubidium(^82^Rb)-PET) and neo-angiogenesis (^68^Ga-NODAGA-RGD) at baseline and one week and four weeks after infarct induction and treatment (Figure 1).

### 2.2. Preparation for Scans

The minipigs were sedated with an intramuscular injection of 0.08 or 0.1 mL/kg (before and after an induced myocardial infarction) of a mixture of zolezapam 10.9 mg/mL and tiletamine 10.9 mg/mL (Zoletil 50/50^®^ vet; Virbac, Carros, France), xylazine 10.9 mg/mL (Xysol vet; ScanVet Animal Health A/S, Fredensborg, Denmark), ketamine 10.9 mg/mL (Ketaminol vet, Intervet International B.V., Boxmeer, the Netherlands), methadone 1.7 mg/mL (Comfortan vet, Dechra Veterinary Products A/S, Uldum, Denmark) and butorphanol 1.7 mg/mL (Torbugesic vet, ScanVet Animal Health A/S, Fredensborg, Denmark). When in lateral recumbency minipigs were moved to the preparation room for instrumentation. For each scan minipigs were instrumented with 3 intravenous accesses (one in each ear (lateral auricular vene) and one in the hind leg (branch of v. saphena)) for anesthesia and PET tracer administration, a urinary bladder catheter, and a tracheal tube for ventilation. During anesthesia saturation (SpO_2_), ECG, non-invasive blood pressure, temperature and end-tidal CO_2_ were monitored using a Datex-Ohmeda S/5 compact monitor (GE Healthcare, Brøndbyvester, Denmark). When fully instrumented the minipigs were transported to the scanner and put on respirator. Respiration was set to obtain normo ventilation with an end-tidal CO_2_ of approximately 55 cmH_2_O. Minipigs were kept anesthetized with a maintenance dose of intravenous propofol 10 mg/kg/h during the ^82^Rb-PET computed tomography (CT) (Siemens mCT, Siemens, 128-slice CT, Knoxville, TN, USA).

### 2.3. Infarct Induction and Stromal Cell Treatment

#### 2.3.1. Göttingen Minipig Adipose-Derived Mesenchymal Stromal Cells

Abdominal subcutaneous adipose tissue was harvested from recently euthanized Göttingen minipigs. The tissue was manually cut from the abdominal region and stored in phosphate-buffered saline (PBS) pH 7.4 (Gibco, Life Technologies, Carlsbad, CA, USA) with 5% penicillin/streptomycin for transportation. The tissue was manually cut into small pieces before being processed as with human samples. The pieces were treated with 0.6 PZ U/mL collagenase NB4 (SERVA Electrophoresis) dissolved in Hank’s buffered saline solution (Gibco, diluted to a concentration of 2 mM Ca^2+^) for one hour at 37 °C. The solution was filtered through a 100 µm filter (Cell Strainer, BD Falcon) and washed trice with PBS. The resulting cells were seeded in T75 flasks in a density of 4.5 × 10^6^ cells/flask in complete medium (alpha-MEM (Gibco, Life Technologies), 10% fetal bovine serum (FBS) (Gibco, Life Technologies), 1% penicillin/streptomycin. The resulting stromal vascular fraction was incubated in an incubator with 37 °C and 5% CO_2_ for 2–3 days after which suspension cells were removed by washing with PBS, before continuing culture. This resulted in a homogeneous population of pASCs, which were stored in liquid nitrogen at a density of 5 × 10^6^ per mL in 5% DMSO (WAK-Chemie Medical, Steinbach, Germany) in FBS. Two weeks before the operation the cells were thawed and cultured in complete medium again. The pASCs used for treatment were in passage 4 or 5.

The cells were tested for mesenchymal stromal cell abilities by performing differentiation towards adipogenic, osteogenic, and chondrogenic lineage using StemPro differentiation kit (Gibco, Life Technologies) according to manufacturer’s protocol. Differentiation was verified by lipid droplets stained with Oil Red O (Sigma-Aldrich, St. Louis, MO, USA) for adipogenic lineage, calcium deposition by Alizarin Red S for osteogenic lineage, and glycosaminoglycans by Alcian Blue 8GX (Sigma-Aldrich, St. Louis, MO, USA) for chondrogenic lineage.

Three minipigs were treated with pASC suspended in alginate hydrogel and two minipigs with saline.

#### 2.3.2. Alginate Hydrogel

The injectable alginate hydrogel was cast using very low viscosity (5cP) high guluronic acid content (G > 65%) alginate (VLVG, NovaMatrix, FMC Biopolymers, Drammen, Norway) dissolved in sterile water. The alginate solution was crosslinked using d-gluconic acid hemi calcium (Sigma-Aldrich, St. Louis, MO, USA) yielding a free-flow injectable alginate hydrogel consisting of 1.5% (*w*/*v*) alginate and 0.9% (*w*/*v*) calcium ions, which was filtered through a 20 µm filter [10]. Centrifuged pASCs were re-suspended in the alginate hydrogel and transferred to a sterile syringe prior to the operation.

#### 2.3.3. Myocardial Infarct Induction and Treatment

The minipigs were sedated, instrumented, ventilated and monitored like described above and kept anaesthetized on propofol (Propofol “B. Braun”; 10 mg/mL; B. Braun Medical A/S, Frederiksberg, Denmark) with a dose range of 10–15 mg/kg/h depending on the individual response to anesthesia. Fentanyl 0.5 µg/kg/h (Fentanyl “Hameln”; 50 µg/mL; Hameln Pharmaceuticals Gmbh, Hameln, Germany) was given as infusion as intraoperative analgesic. Amoxicillin 15 mg/kg (Curamox Prolongatum Vet.; 150 mg/mL; Boehringer Ingelheim DK A/S, Kalundborg, Denmark) and meloxicam 0.4 mg/kg (Boehringer Ingelheim DK A/S, Denmark) were given prophylactic before operation. Before surgical incision 50 mg of amiodaronhydrochlorid was administered intravenously as a bolus. The heart was accessed through partial lower median sternotomy. A sternal retractor was inserted, and the pericardium was opened with a scissor. The left anterior descending artery and a large diagonal branch were identified. Myocardial infarction was induced by ligation with a 5-0 prolene suture of the apical part of the LAD artery or a large diagonal artery (D2). After ligation of the artery, a sharp demarcation line between ischemic and non-ischemic myocardium was observed. Depending on the treatment arm, either isotonic saline or pASCs in alginate hydrogel was injected in 4–6 injections with a 25 G needle in the peri-infarct area on one side of the infarction. The minipigs in the treatment group received approximately 40 × 10^6^ pASCs in 2.5 mL alginate hydrogel. Afterward, the sternum was closed with PDS 0 suture, the fascia and subcutis with 0 vicryl, and the cutis with a 4-0 monocryl intradermal suture. The minipigs were treated post-operative with butorphanole 0.2 mg/kg (Torbugesic vet 10 mg/mL, ScanVet Animal Health A/S, Denmark) and methadone 0.2 mg/kg (Comfortan vet 10 mg/mL, Dechra Veterinary Products A/S, Denmark) every 3–4 h for the first 24 h. Subsequently, they were treated with buprenorfin 0.01–0.02 mg/kg (Vetergesic 0.3 mg/mL, Orion Pharma Animal Health, Copenhagen, Denmark) every 8th hour in 2–3 days. The opioid regime was supported by oral administration of meloxicam 0.4 mg/kg (metacam oral suspension 15 mg/mL, Boehringer Ingelheim DK A/S) for 5 days post-operative. Antibiotic treatment with amoxicillin 20 mg/kg (Clamoxyl VET. 51%, Orion Pharma Animal Health, Denmark) was continued orally in 6 days post-operative.

### 2.4. Radiochemistry: ^68^Ga-NODAGA-E[c(RGDyK)]_2_ Synthesis

NODAGA-E[c(RGDyK)]_2_ acetate was obtained from ABX GmbH (Radeberg, Germany). Gallium-68 (t_1/2_ = 68 min; E_max_,_β+_ = 1.90 MeV (89%)) labelling of NODAGA-E[c(RGDyK)]_2_ acetate was performed using a Modular-Lab eazy module (Eckert & Ziegler, Berlin, Germany). The ^68^Ge/^68^Ga generator (IGG100, Eckert & Ziegler) was eluted with 6 mL 0.1 M HCl. The eluate was concentrated on a Bond Elut SCX cartridge and eluted with 600 µL 5 M NaCl/5.5 M HCl (41:1). NODAGA-E[c(RGDyK)]_2_ (30 nmol) was labelled in 1000 µL 0.7 M NaOAc buffer pH 4.5 and 400 µL 50% EtOH at 60 °C for 400 s. The resulting ^68^Ga-NODAGA-E[c(RGDyK)]_2_ was formulated with saline or phosphate buffer.

The radiochemical purity was more than 96% on HPLC, and the amount of unlabeled ^68^Ga in the product was less than 1%, as demonstrated by radio–thin layer chromatography.

All reagents and cassettes were purchased from Eckert & Ziegler. For analysis, a high-performance liquid chromatograph (Ultimate 3000; Dionex, Sunnyvale, CA, USA) was used with a 2.6-μm, 100-Å, 50 × 4.6 mm C18 Kinetex column (Phenomenex, Torrance, CA, USA). The mobile phases were: eluent A: 10% MeCN in H_2_O with 0.1% trifluoroacetic acid; eluent B: 10% H_2_O in MeCN with 0.1% trifluoroacetic acid.

### 2.5. ^82^Rubidium and ^68^GA-NODAGA-RGD PET Imaging

^82^Rb rest and stress myocardial perfusion PET-CT were performed before induction of myocardial infarction, one week and one month after infarction (Figure 1). The ^68^GA-NODAGA-RGD (RGD) PET-CT was performed as a 10 min ECG-gated scan 45 min after administration of 100 MBq RGD. The ^82^Rb rest and stress myocardial perfusion PET-CT has previously been described in detail [11,12]. In brief, a 7 min dynamic PET myocardial perfusion rest scan under administration of 1000–1200 MBq ^82^Rb was performed. Subsequently, a 7 min dynamic dipyridamole stress PET scan was performed. Dipyridamole (140 µg/kg/min) was given as a continuous intravenous infusion over 4 min prior to ^82^Rb-tracer injection which followed 3–5 min after the completion of dipyridamole infusion. PET images were analyzed semi-automatically using Cedars-Sinai Cardiac Suite QPS/QGS^®^ (Cedars-Sinai Medical Center, Los Angeles, CA, USA) for Syngo.Via (Siemens, Knoxville, TN, USA). The accuracy of slice-alignments in the ventricle was assessed by planes and intervened if necessary. Perfusion defects were subsequently quantified in the total left ventricle myocardium. The magnitude of the rest perfusion defects was determined automatically by comparing the polar plot of a minipig to that of the human normal database on a pixel-by-pixel basis. A 2.5 standard deviation cut-off was used to define whether a pixel count fell below a normal value. Mean Standardized Uptake Values were measured in the infarcted area (SUV_mean,infarct_) as well as the non-infarcted myocardium including blood pool (SUV_mean,background_) and the ratio between the two (SUV_index_) was calculated.

During the examination heart rate was measured continuously and non-invasive blood pressure was measured every minute. The hemodynamic response to dipyridamole was evaluated by calculating Rate-Pressure-Products (RPP) at rest and stress as the product of non-invasive systolic blood pressure and heart rate at rest and stress, respectively.

### 2.6. Histology

After euthanasia, the heart of the minipig was removed and the infarct area was located together with the suture. The heart was cut into 1.5 cm thick short axis slices, and 4 areas from the slice below the ligation were selected for histology. The four areas were: (1) Infarct area; (2) Peri-infarct area; (3) Peri-infarct area at the side of injections; and (4) Remote myocardium, beyond the peri-infarct area. The tissue was fixed in 4% paraformaldehyde and embedded in paraffin. Slices of 5 µm thickness were cut for staining at four different levels in each tissue sample. Antigen retrieval was performed before all stainings. The tissue was stained for collagen by Masson’s Trichrome (Hospital Pharmacy, Rigshospitalet), tissue organization and inflammation by hematoxylin and eosin (H&E) (Hospital Pharmacy, Rigshospitalet), macrophages by CD68 (Agilent Technologies, Glostrup, Denmark, 1:100), myofibroblasts by α-smooth muscle actin (α-SMA) (Agilent Technologies), CD31 (Bio-Rad Laboratories, Copenhagen, Denmark, 1:50), and integrin α_v_β_3_ (Merck Millipore, Darmstadt, Germany, 1:75). Visualization was performed used Carl Zeiss Axio Imager Z.1 microscope and Axiovision 4.6.3 software. Mason’s Trichrome was analyzed by three random areas at ×1.25 magnification for each slice, resulting in a total of 12 random fields for each heart area from each minipig. The percentage of collagen staining compared to the whole image was analyzed using Image J software (Fiji). A similar number of fields were acquired for each staining. The level of inflammation was assessed by scoring each ×5 magnification field from 0 to 3, ranging from 0 = no visible inflammation, 1 = <10% inflammatory cells, 2 = 10–50% inflammatory cells, and 3 = >50% inflammatory cells of total cells. High power fields of ×20 magnification were used for total manual CD68 count. Similarly, the same magnification was used for α-SMA fibroblast percentage measurements in the scar area, and CD31 and integrin α_v_β_3_. All manual assessment was performed by two independent observers. 

### 2.7. Statistical Analysis

Categorical variables were expressed as percentages and continuous variables were reported as means and standard deviations. Differences in continuous variables between groups were assessed with students *t*-test while differences in groups over time were assessed with paired *t*-test. A general linear model was used to analyse data with more than one time-point and the resulting regression lines were compared for differences between groups. A two tailed *p*-value < 0.05 was considered statistically significant. Statistical analyses were performed using SAS^®^ for Windows, version 9.1 (SAS institute, Cary, NC, USA).

## 3. Results

### 3.1. ^82^Rubidium PET before Myocardial Infarction

Before induction of myocardial infarction all minipigs showed a homogenic distribution of ^82^Rb in both rest and stress ^82^Rb PET images and no RGD uptake was noted. During stress, RPP doubled from about 5000 at rest to about 10,000 mmHg/min, mainly due to increased heart rate. Mean CFR was 2.80 (±1.17) for non-pASC-treated minipigs and 2.25 (±0.43) for pASC-treated minipigs. Mean ejection fractions (EF) were 80 (±4)% and 92 (±2)% during rest and 87 (±8)% and 91 (±1)% during dipyridamole stress in non-pASC and pASC-treated minipigs (Table 1).

### 3.2. ^82^Rubidium PET after Myocardial Infarction

**Figure 2:**
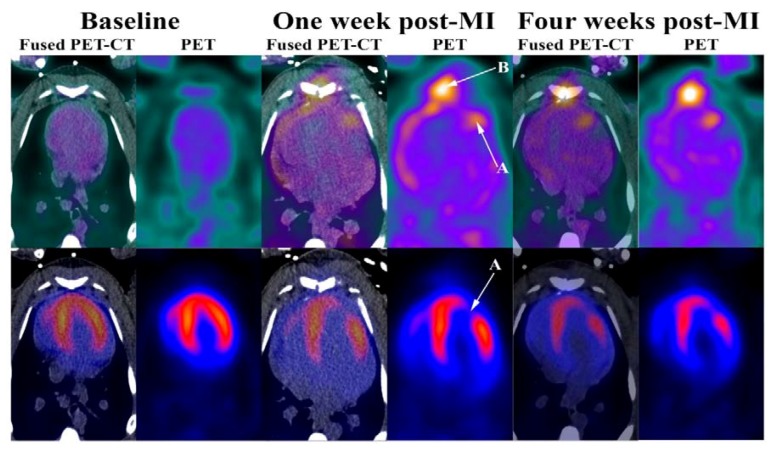
Modified from [12]. RGD (**Top**) and ^82^Rb stress (**Bottom**) PET at baseline, one week and one month after induced myocardial infarction. (**A**) Myocardial infarction; (**B**) Sternotomy.

One week and one month after myocardial infarction, four of the five minipigs (two stem-cell-treated and two non-stem-cell-treated) showed a myocardial perfusion defect located to the anterior ventricle myocardium (Figure 2). One minipig, with confirmed myocardial infarction after one week, died before the planned one-month post infarction scan. Characteristics in pASC-treated and saline-treated minipigs at baseline, one week and one month following myocardial infarction are provided in Table 1. Myocardial stress flows were significantly lower at follow-up compared to baseline in non-pASC-treated minipigs (1.80 (±0.20) mL/g/min vs. 1.30 (±0.16) mL/g/min, *p* < 0.05), something that was not noted in pASC-treated minipigs. Even though, there was no difference in the decrease of stress flow or coronary flow reserve from baseline to follow-up between non-pASC vs. pASC-treated minipigs when comparing regression lines. In pASC-treated minipigs end diastolic volumes (EDV) tended to be higher at follow-up compared to baseline at rest (25 (±3) mL vs. 39 (±6) mL, *p* = 0.06) and were significantly higher at stress (20 (±2) mL vs. 33 (±1) mL, *p* < 0.05), something that was not observed in non-pASC-treated minipigs (Table 1). Left ventricular ejection fractions (LVEF), on the other hand, did not change from baseline to follow-up in either non-pASC-treated (80 (±4)% vs. 81 (±5)% at rest, and 87 (±8)% vs. 89% at stress) or pASC-treated minipigs (92 (±2)% vs. 85 (±8)% at rest, and 81 (±3)% vs. 89 (±3)% at stress) (Table 1).

### 3.3. ^68^NODAGA-RGD PET after Myocardial Infarction

All minipigs showing myocardial perfusion defects in the ^82^Rb PET images also showed RGD uptake in the infarcted myocardium one week and one month after myocardial infarction regardless of pASC treatment or not (Figure 1). SUV_mean_ in the infarcted myocardium was significantly higher than the background SUV_mean_ (non-infarcted myocardium including blood pool) after both one week and one month (*p* < 0.05 for both). Mean SUV_mean_ in the RGD uptaking myocardium in pASC-treated minipigs was 1.23 (±0.40) and 1.02 one week and one month after infarction while SUV_mean_ was 1.44 (±0.13) and 1.26 (±0.01) in saline-treated minipigs (non-significant). The index between SUV in the infarcted and background (SUV_index_) was 1.37 (±0.22) and 1.19 one week and one month after infarction in pASC-treated minipigs (*p*-value could not be calculated because only one minipig was alive at the last scan) and 1.34 (±0.05) and 1.22 (±0.06) for saline-treated minipigs (*p* < 0.05) (Table 2). Thus, SUV_index_ in saline-treated minipigs one month after infarction were significantly lower than SUV_index_ one week after infarction. There was no difference in SUV_index_ between pASC-treated vs. saline-treated minipigs after one week, whereas groups could not be compared for differences after one month due to only one pASC-treated minipig left with confirmed myocardial infarction.

### 3.4. Histological Findings

**Figure 3:**
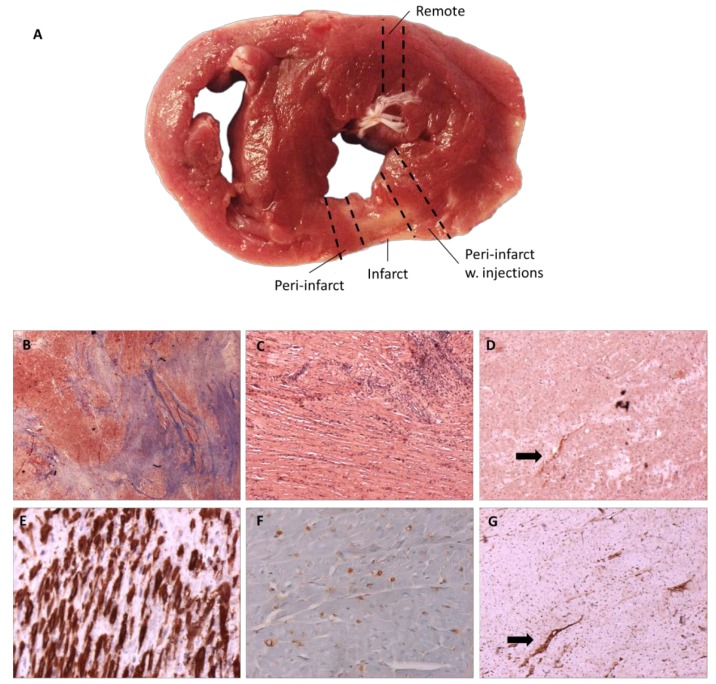

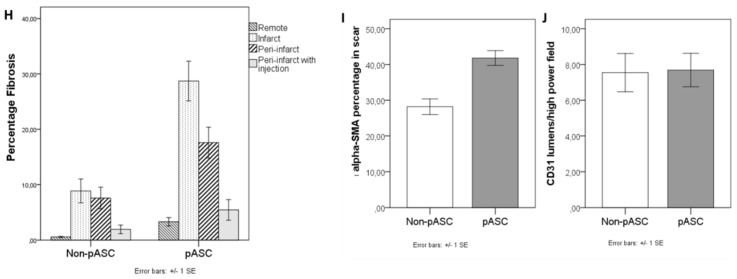
Myocardial histology. (**A**) Representative image of histology sampling from infarcted areas (**B**,**C**,**E**); peri-infarcted areas (**D**,**F**,**G**); (**B**) Masson’s Trichrome ×1.25 (**C**) Hematoxylin-eosin ×5 (**D**) Integrin α_v_β_3_ ×20 (**E**) α-smooth muscle actin ×20 (**F**) CD68 ×20 (**G**) CD31 ×20 (**H**) Percentage fibrosis in all sample areas (I) α-smooth muscle actin percentage in scar (**J**) CD31 vessel density per high power field. pASC: porcine adipose-derived stromal cells in alginate hydrogel, Arrows show co-localization of integrin α_v_β_3_ and CD31.

During dissection, a small but clearly infarcted area was visible in all minipigs except one, confirming the ^82^Rb perfusion imaging. For comparison purposes, the minipig that died prematurely was also included in the histology analysis. Integrin α_v_β_3_ was identified mainly in the infarcted area, and expression was co-localized with CD31 on adjacent slices (Figure 3). No difference in CD31 positive vessel density was observed between the groups. Similarly, no difference was observed between the level of inflammation and CD68 positive cells. There was a tendency of increased collagen tissue and scar area occupied by α-SMA positive myofibroblasts in the pASC group. 

## 4. Discussion

The major finding of this study was that infarcted myocardium in minipigs had significantly higher RGD uptake after one week and one month, whether pASC-treated or non-pASC-treated. Overall, SUV_index_ tended to be lower one month after infarction compared to one week after infarction (*p* = 0.10) (not shown). Furthermore, we found no difference in SUV_index_ between pASC-treated vs. non-pASC-treated minipigs, which also was reflected in the histological findings of similar CD31 vessel density in the groups, with co-localization of integrin α_v_β_3_ and CD31.

Our finding of integrin α_v_β_3_ only in the infarcted area is in line with a recent study (Grönman et al.), using the same RGD peptide, but not the same tracer, to detect angiogenesis in pigs after induction of coronary stenosis [13]. They found integrin α_v_β_3_ and their tracer almost exclusively located in the injured myocardium.

In addition to this, we found no differences in ejection fractions or coronary flow reserves between pASC-treated vs. non-pASC-treated minipigs. This relates to the similar CD31 vessel density observed by histology. There was a tendency for a greater degree of fibrosis and a higher density of α-SMA expression in the scar of the pASC-treated pigs. The difference was apparently too small to be functionally relevant. It has been shown, that the effect of stromal cell therapy is dependent on the extent of the injury, with more injury resulting in more pronounced treatment effect [14]. Hence, the relatively small infarcts could be the reason for the observed outcome. 

In a study by Cai et al., the angiogenic effect of vascular endothelial growth factor (VEGF) gene and/or bone marrow mesenchymal stem cells (BMSCs) following myocardial infarction was compared to a saline control, using Spraque–Dawley rats (*n* = 4 in each group) [15]. The angiogenic response was evaluated using ^18^F-alfatide II (^18^F-AlF-NOTA-E[PEG_4_-c(RGDfk)]_2_), which, as RGD, targets α_v_β_3_ integrin. Corresponding to our findings, RGD uptake was documented in all four groups one week after myocardial infarction. The infarct-to-normal myocardium ratio was considerably higher in all groups (3.94 ± 0.20 for VEGF group, 3.77 ± 0.16 for BMSCs group and 4.86 ± 0.08 for the combination group vs. 3.01 ± 0.03 for the control group) compared to our study. Contrary to our findings, the study documented an angiogenic effect of treatment in all treatment groups compared to the control group (*p* < 0.005 for VEGF group, *p* < 0.005 for BMSCs group and *p* < 0.0001 for the combination group). Whether the differences in infarct to normal heart ratios rely on the different tracers used, differences in species or infarct size and whether the different conclusions rely on our relatively small sample size remains unsettled.

In another study angiogenesis after myocardial infarction by ligation of LAD in Spraque–Dawley rats using [^68^Ga]DOTA-E-[c(RGDfK)]_2_ PET was examined [16]. The infarcted myocardium showed higher tracer uptake than the remote myocardium 7 days and 4 weeks post-infarct (infarct/remote myocardium ratio 2.25 (±0.14) and 2.13 (±0.37)). Compared to sham-operated-rats both infarcted and remote myocardium showed higher tracer uptake 7 days and 4 weeks postinfarct where it coincided with increased interstitial fibrosis. Again, whether the higher infarct/remote myocardium ratio compared to the ones measured in our study relies on tracer difference, species or infarct size remains unknown.

The primary outcomes for most clinical trials investigating the effect of cellular therapy are LVEF, EDV or end-systolic volume (ESV) measured by MRI, echocardiography, and/or CT. These are fine as surrogate endpoints for phase I/II clinical trials but do not relate directly to the mechanisms by which the cells exert their regenerative effect. The addition of other endpoints might clarify the effect of cell therapy [17,18].

Angiogenesis is a vital part of the reparative process following myocardial infarction. It is also thought to be one of the main mechanisms behind the regenerative effect of cardiac cellular therapy and is in pre-clinical studies traditionally assessed in histology by counting of vessel density in affected areas of the heart [19,20,21]. Since this is not feasible in clinical studies, clinical trials with thorough functional design do not usually investigate angiogenesis due to lack of methods [22]. While angiogenesis is associated with post-infarct remodeling of the left ventricle, stratification of patients with RGD-PET following myocardial infarction may prove effective [2]. Although strictly hypothetical, one would assume a patient with RGD uptake to have a better prognosis than a patient not taking up RGD in a myocardial infarction of a similar size. Furthermore, the “clearance” of RGD at follow-up PET might be of importance. Indicative of the latter, although limited to two myocardial infarction patients, was a study by Luo et al. [23]. Both patients showed retention of ^68^Ga-BNOTA-PRGD_2_ in the infarcted region of the myocardium 1 week after the cardiac event. At follow-up, retention of ^68^Ga-BNOTA-PRGD_2_ remained in the infarcted myocardium of the still symptomatic patient whereas no uptake could be visualized in the asymptomatic patient. Further studies are required to clarify the association between RGD uptake and post-infarct prognosis. Moreover, the most favorable time point for investigation of RGD uptake post infarct remains unsettled.

Interpretation of RGD uptake is not limited to ischemic heart disease; post-stroke repair processes after ischemic injury are subject to neurovascular research. Tumor angiogenesis has been well recognized as an essential hall mark of tumor growth, invasion and metastasis and, therefore, RGD PET can be used for tumor detection and staging in different types of cancer to provide additional information to existing imaging modalities [24,25]. A number of other RGD peptide PET tracers have been clinically investigated including ^18^F-Galacto-RGD, ^18^F-Fluciclatide, ^18^F-RGD-K5, ^18^F-FPPRGD_2_, ^18^F-Alfatide, ^68^Ga-NOTA-RGD and ^68^Ga-NOTA-PRGD_2_ [25]. The detection efficiency of lesions in different regions is highly dependent on the difference in in vivo biodistribution patterns of each single RGD peptide.

## 5. Limitations

Our relatively small number of animals in each group was a limitation, together with the relatively small myocardial infarctions. Another limitation was that the specificity of RGD was not studied by a non-targeted control tracer or blocking experiments. As a result, we were limited to conclude that (1) RGD uptake was located specifically to the myocardial infarct area; (2) histologically, integrin α_v_β_3_ was only found in the infarct area and not in remote areas; (3) histologically, integrin α_v_β_3_ was co-localized with CD31. In summary, the increased RGD uptake in the myocardial infarct area was a strong indicator of angiogenesis.

## 6. Conclusions

Myocardial infarctions were verified by ^82^Rb dipyridamole stress PET. Indications of angiogenesis in the myocardial infarct area were documented by increased RGD uptake located specifically to the MI area in RGD-PET which, in addition, was the only area that histologically showed presence of integrin α_v_β_3_ co-localized with CD31. However, no differences between pASC-treated minipigs and non-pASC-treated minipigs could be shown.

## Figures and Tables

**Table 1:** Left ventricle function parameters and perfusion before and after induction of a myocardial infarction in pASC and non-pASC-treated minipigs measured by ^82^Rb PET-CT.

	Non-pASC Treatment				pASC Treatment			
	Baseline (*n* = 2)	1 Week Post-MI (*n* = 2)	4 Weeks Post-MI (*n* = 2)	*p*-Value	Baseline (*n* = 3)	1 Week Post-MI (*n* = 3)	4 Weeks Post-MI (*n* = 2)	*p*-Value
Rest flow (mL/g/min)	0.73 (±0.19)	0.92 (±0.08)	0.70 (±0.16)	NS	0.94 (±0.19)	1.02 (±0.58)	1.38 (±0.76)	NS
Rest EDV (mL)	30 (±3)	26 (±4)	31 (±6)	NS	25 (±3)	35 (±8)	39 (±6)	0.06
Rest ESV (mL)	6 (±1)	5 (±1)	6 (±3)	NS	2 (±0)	6 (±3)	6 (±2)	NS
Rest EF (%)	80 (±4)	84 (±1)	81 (±5)	NS	92 (±2)	84 (±4)	85 (±8)	NS
Stress flow (mL/g/min)	1.80 (±0.20)	1.61 (±0.08)	1.30 (±0.16)	<0.05	2.07 (±0.23)	1.80 (±0.42)	2.05 (±0.36)	NS
Stress EDV (mL)	29 (±4)	25 (±1)	26 *	NS	20 (±2)	27 (±7)	33 (±1)	<0.03
Stress ESV (mL)	4 (±2)	5 (±1)	3 *	NS	2 (±1)	5 (±1)	4 (±1)	NS
Stress EF (%)	87 (±8)	83 (±2)	89 *	NS	91 (±3)	82 (±1)	89 (±3)	NS
CFR	2.80 (±1.17)	1.79 (±0.26)	1.95 (±0.29)	NS	2.25 (±0.43)	2.10 (±0.96)	1.68 (±0.67)	NS

Abbreviations: pASC: porcine adipose-derived mesenchymal stromal cell; EDV: End-diastolic-volume; ESV: End-systolic-volume; EF: Ejection fraction; CFR: Coronary flow reserve; MI: Myocardial infarction. *: Only one measurement was possible, due to ECG trigger problems; NS: Non-significant.

**Table 2:** (**A**) RGD uptake in non-pASC-treated minipigs with myocardial infarction. (**B**) RGD uptake in pASC-treated minipigs with myocardial infarction.

	One Week Post Infarction	Four Weeks Post Infarction	*p*-Value
(**A**) non-pASC-treated minipigs			
SUV_Mean,infarct_	1.44 (±0.13)	1.26 (±0.01)	NS
SUV_Mean,background_	1.07 (±0.06)	1.04 (±0.06)	NS
SUV_index_	1.34 (±0.05)	1.22 (±0.06)	<0.05
(**B**) pASC-treated minipigs			
SUV_Mean,infarct_	1.23 (±0.40)	1.02 *	-
SUV_Mean,background_	0.88 (±0.16)	0.86 *	-
SUV_index_	1.37 (±0.22)	1.19 *	-

Abbreviations: pASC: porcine adipose-derived mesenchymal stromal cell; SUV_Mean,infarct_: Standardized Uptake Value in infarcted myocardium; SUV_Mean,background_: Standardized Uptake Value in non-infarcted myocardium including blood pool; SUV_index_: (Standardized Uptake Value in infarcted myocardium)/(Standardized Uptake Value in non-infarcted myocardium including blood pool). *: Only one minipig with proven MI.
